# Gut microbiota modulation induced by Zika virus infection in immunocompetent mice

**DOI:** 10.1038/s41598-020-80893-y

**Published:** 2021-01-14

**Authors:** Rafael Corrêa, Igor de Oliveira Santos, Heloísa Antoniella Braz-de-Melo, Lívia Pimentel de Sant’Ana, Raquel das Neves Almeida, Gabriel Pasquarelli-do-Nascimento, Paulo Sousa Prado, Gary P. Kobinger, Corinne F. Maurice, Kelly Grace Magalhães

**Affiliations:** 1 Laboratory of Immunology and Inflammation, Department of Cell Biology, University of Brasilia, Brasília, DF Brazil; 2 Central Laboratory of Federal District (LACEN), Brasília, Brazil; 3 Département de Microbiologie-Infectiologie et d’Immunologie, Université Laval, Quebec, Canada; 4 Centre de Recherche en Infectiologie du CHU de Québec-Université Laval, Quebec, Canada; 5 Department of Microbiology and Immunology, McGill University, Montreal, Canada

**Keywords:** Microbiome, Immunology, Microbiology

## Abstract

Gut microbiota composition can modulate neuroendocrine function, inflammation, and cellular and immunological responses against different pathogens, including viruses. Zika virus (ZIKV) can infect adult immunocompetent individuals and trigger brain damage and antiviral responses. However, it is not known whether ZIKV infection could impact the gut microbiome from adult immunocompetent mice. Here, we investigated modifications induced by ZIKV infection in the gut microbiome of immunocompetent C57BL/6J mice. Adult C57BL/6J mice were infected with ZIKV and the gut microbiota composition was analyzed by next-generation sequencing of the V4 hypervariable region present in the bacterial 16S rDNA gene. Our data showed that ZIKV infection triggered a significant decrease in the bacteria belonging to Actinobacteria and Firmicutes phyla, and increased Deferribacteres and Spirochaetes phyla components compared to uninfected mice. Interestingly, ZIKV infection triggered a significant increase in the abundance of bacteria from the Spirochaetaceae family in the gut microbiota. Lastly, we demonstrated that modulation of microbiota induced by ZIKV infection may lead to intestinal epithelium damage and intense leukocyte recruitment to the intestinal mucosa. Taken together, our data demonstrate that ZIKV infection can impact the gut microbiota composition and colon tissue homeostasis in adult immunocompetent mice.

## Introduction

Zika Virus (ZIKV) is an Arbovirus member of the Flaviviridae family that is mainly transmitted by the bite of *Aedes* genus mosquitoes^1^. During the 2015 outbreak, ZIKV spread quickly in America, mainly in Brazil^2^. During ZIKV dissemination, non-vector born routes of infection were reported, including sexual transmission^3^. A great concern about ZIKV's impact worldwide was the association of post-infection disorders, such as Guillain-Barré syndrome^4^, and the development of congenital malformations^5^.

As a means of better understanding ZIKV pathogenesis, most of the molecular mechanisms associated with the infection were elucidated with the use of genetically-induced immunodeficient mice models^6^. In those reports, infected mice presented weight loss, high viremia, detectable signs of illness, intensive neuronal loss, immune system activation on neuronal surroundings, and severe testicular inflammation^6–8^. In contrast, the impact of ZIKV on immunocompetent adult mice models showed detectable viral loads in the serum, and effects on different organs or lethality after infection through different routes^9,10^. Despite this, ZIKV induces innate and adaptive immune responses that are essential for protecting the organism against the establishment of disease^11,12^. The immune system activation demonstrates that specific parameters are modulated during ZIKV infection such that, regardless of the milder symptoms, the virus still impacts on the host^13^. These findings lead to new questions regarding if other alterations could be linked to viral activation of the host’s immune system and modulation of physiological functions not noticed before.

The gut microbiota has been described as a strong modulator of inflammatory and immune responses, both locally and systemically^14,15^, playing essential roles in triggering host responses against pathogen infections^16^. In recent years, studies have shown that the gut microbiota influences and is influenced by viral infections^17^. This study reports that enteric viruses can lead to substantial disturbances in gut microbiota composition, impacting on parameters of viral infectivity and, as a consequence, the host’s health^17^. However, the effects of diseases induced by other non-enteric viruses on the gut microbiota are still poorly understood. Thus, it is not known whether ZIKV can change the gut microbiota composition of immunocompetent mouse models after infection. This work aims to characterize the gut microbiota of an immunocompetent mouse model after ZIKV infection as a means of providing new perspectives regarding the interactions between the intestinal microbial community and non-enteric viral infections.

## Results

### ZIKV infection changes the β-diversity of the gut microbiota of mice

**Figure 1:**
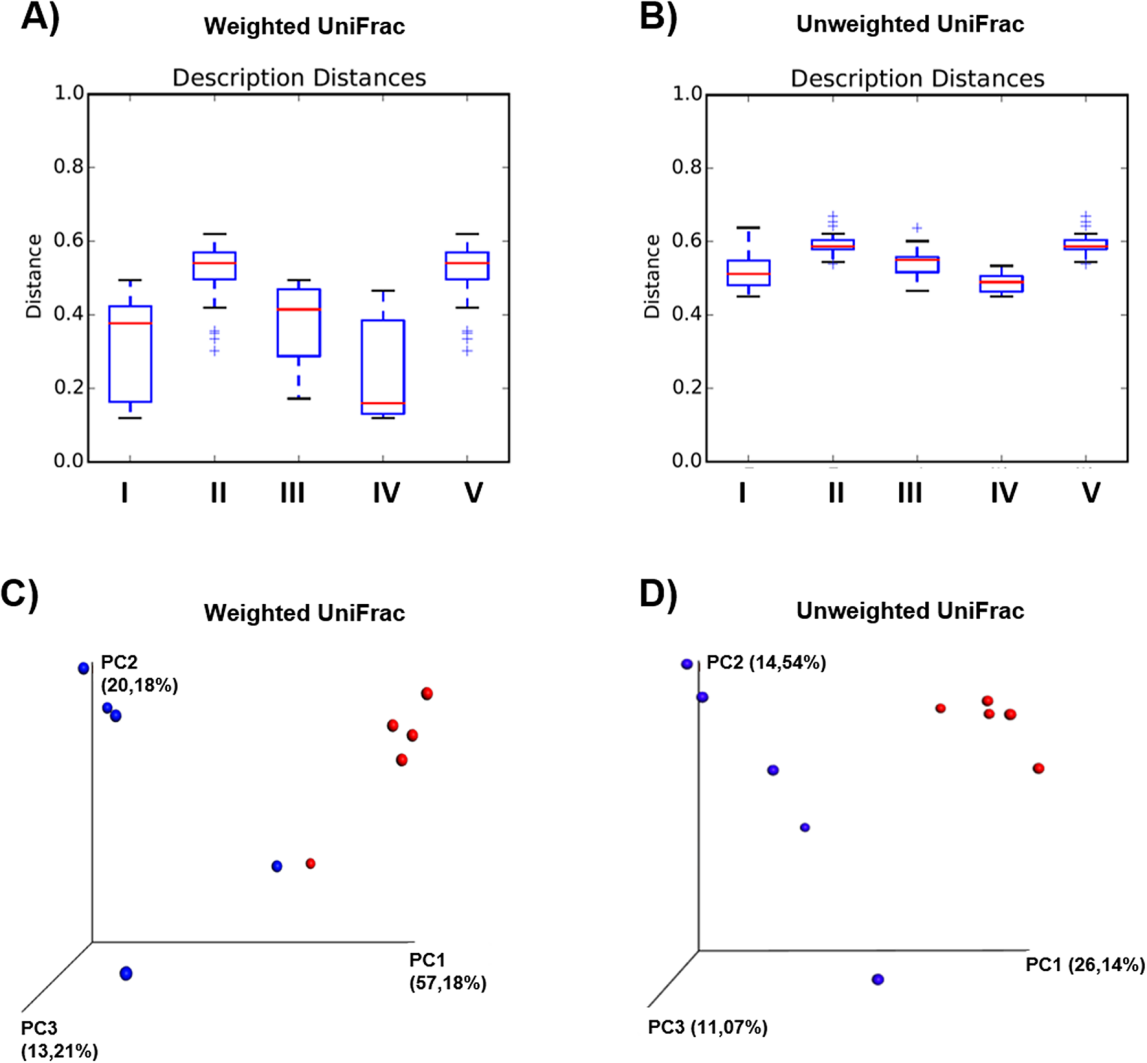
β-diversity analysis of the gut microbiota between ZIKV-infected and uninfected mice. Distance boxplots of weighted (**A**) and unweighted Unifrac (**B**): I—All within the description, II—All between description, III—Uninfected vs. Uninfected, IV—ZIKV vs. ZIKV, and V—Uninfected vs. ZIKV. (**C**) PCoA plot weighted Unifrac and (**D**) PCoA plot unweighted Unifrac, blue: Uninfected, red: ZIKV. Significance was tested using a two-sided Student’s two-sample t-test; the non-parametric p-values were calculated with Bonferroni correction. p-values for comparisons of Distance boxplots (**A**): I vs. II p = 0.010, I vs. III p = 1, I vs. VI p = 1, I vs. V p = 0.010, II vs. III p = 0.010, II vs. IV p = 0.010, II vs. V p = 1, III vs. IV p = 0.420, III vs. V p = 0.010, and IV vs. V p = 0.010; (**B**): I vs. II p = 0.010, I vs. III p = 1, I vs. VI p = 1, I vs. V p = 0.010, II vs. III p = 0.010, II vs. IV p = 0.010, II vs. V p = 1, III vs. IV p = 0.080, III vs. V p = 0.030, and IV vs. V p = 0.010.

Alpha diversity refers to the diversity of microbial communities within a single particular condition (infected or uninfected), while beta diversity describes the difference in microbial communities between conditions (comparison between groups)^18^. Using Qiime^19^, we quantified gut microbial diversity within each group (α-diversity) of uninfected and ZIKV-infected immunocompetent mice and characterized the gut bacterial diversity induced by ZIKV infection in comparison to the uninfected group (Supplementary Fig. 1). The analysis of composition variation of bacterial species between the different groups of mice (β-diversity) provided a measure of distance and dissimilarity between samples. Beta diversity can be classified into quantitative (weighted UniFrac) or qualitative (unweighted UniFrac) indices. We verified that ZIKV infection induced a sample clustering of the infected group, showing that after infection there is a decrease in the distance between the samples (i.e. they are more similar). We also characterized the phylogenetic proximity between the gut microbiota from samples in the quantitative analysis, weighted UniFrac (Fig. 1A) and the qualitative analysis, shown by the unweighted UniFrac data (Fig. 1B).

Observations based on PCoA plots can be substantiated with statistical analyses that assess the clusters. Principal coordinates (PCs) explain a certain fraction of the variability, observed in the data set, and are plotted to create a visual representation of the microbial community compositional differences among samples^18^. The PC values of weighted Unifrac (Fig. 1C) were higher compared to the PC values of unweighted Unifrac (Fig. 1D), which suggests that relative abundance is more important than taxonomic richness. Focusing on weighted Unifrac, which takes into account the relative abundance of bacterial taxa, thus limiting the impact of low abundance bacteria, we observed that sample clustering of gut microbial communities is more evident in the ZIKV-infected mice, with a smaller distance value (Fig. 1A) and higher principal coordinate values (Fig. 1C).

### ZIKV infection decreases the abundance of Actinobacteria and Firmicutes in the gut microbiota

**Figure 2:**
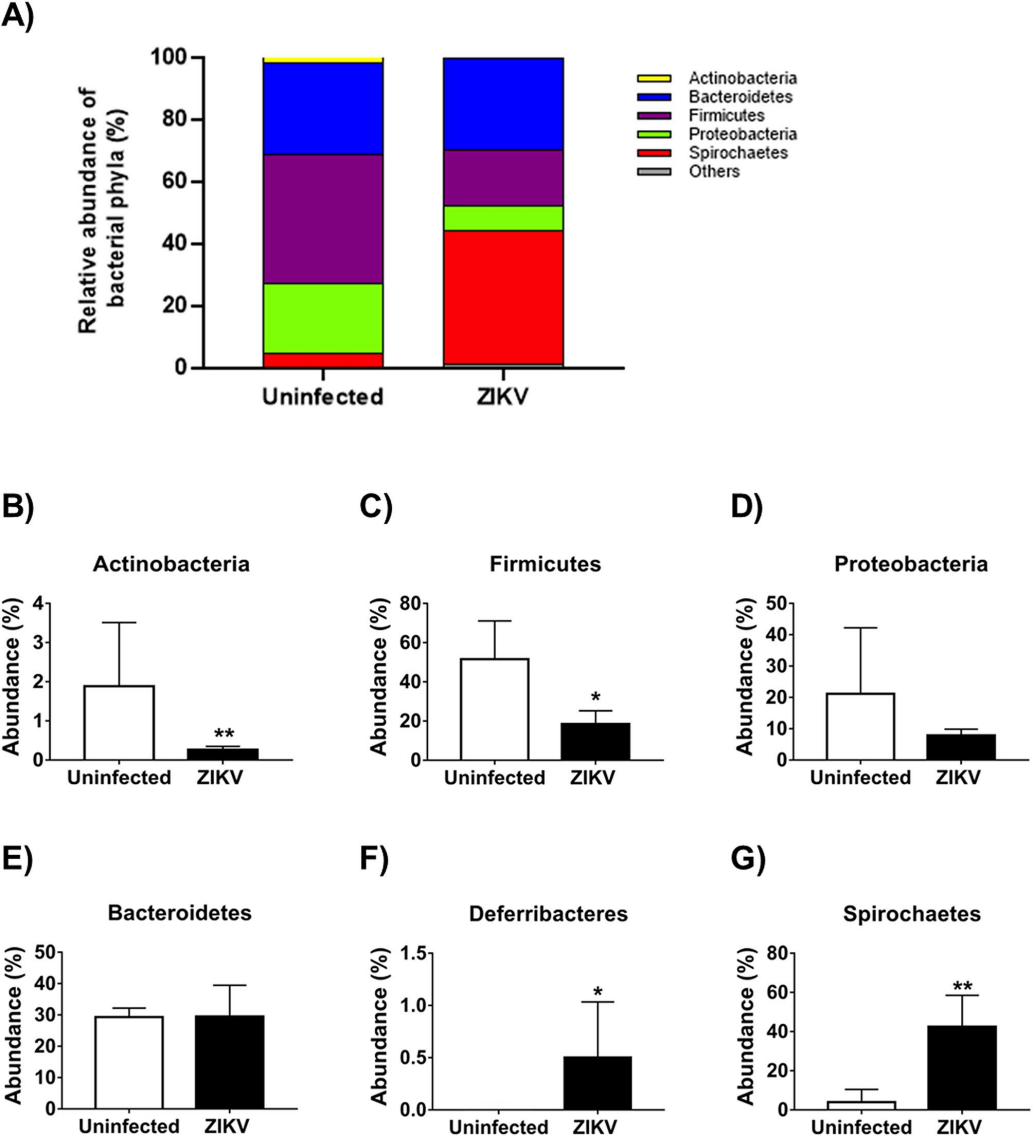
Phylum analysis of the gut microbiota modulation induced by ZIKV infection in the adult immunocompetent mice. C57BL/6J mice (N = 5) were infected with ZIKV and fecal samples were collected after 14 days post-infection. The gut microbiota diversity was assessed by 16S rDNA gene sequencing of the V4 region using the Illumina HiSeq platform. Bar charts represent the relative abundance of Phylum (**A**), Actinobacteria (**B**), Firmicutes (**C**), Proteobacteria (**D**), Bacteroidetes (**E**), Deferribacteres (**F**), and Spirochaetes (**G**). Statistical analyses were performed using a Student’s two-sample t-test, the non-parametric p-values were calculated with the Mann Whitney test, the bars represent a confidence interval of 95% (*p < 0.05; **p < 0.01).

The taxonomic analysis identified Actinobacteria, Bacteroidetes, Firmicutes, Proteobacteria, and Spirochaetes as the most abundant phyla in both ZIKV-infected and uninfected mice (Fig. 2A). In the ZIKV-infected group, the abundance of Actinobacteria significantly decreased from 1.8 to 0.3%, (Fig. 2B), and Firmicutes significantly decreased from 41.8 to 18.2% (Fig. 2C) relative to the uninfected controls. In contrast, ZIKV infection increased the relative abundance of Deferribacteres from undetected to 0.5% (Fig. 2F) and Spirochaetes from 4.2 to 42.9% (Fig. 2G) when compared to uninfected mice. The relative abundances of Bacteroidetes (Fig. 2E) and Proteobacteria (Fig. 2D) were not modulated by ZIKV infection. Thus, ZIKV infection triggered a significant change in bacterial community composition in the immunocompetent mice at the phylum level.

### ZIKV infection increases the abundance of the Deferribacteraceae and Spirochaetaceae families in the gut microbiota

**Figure 3:**
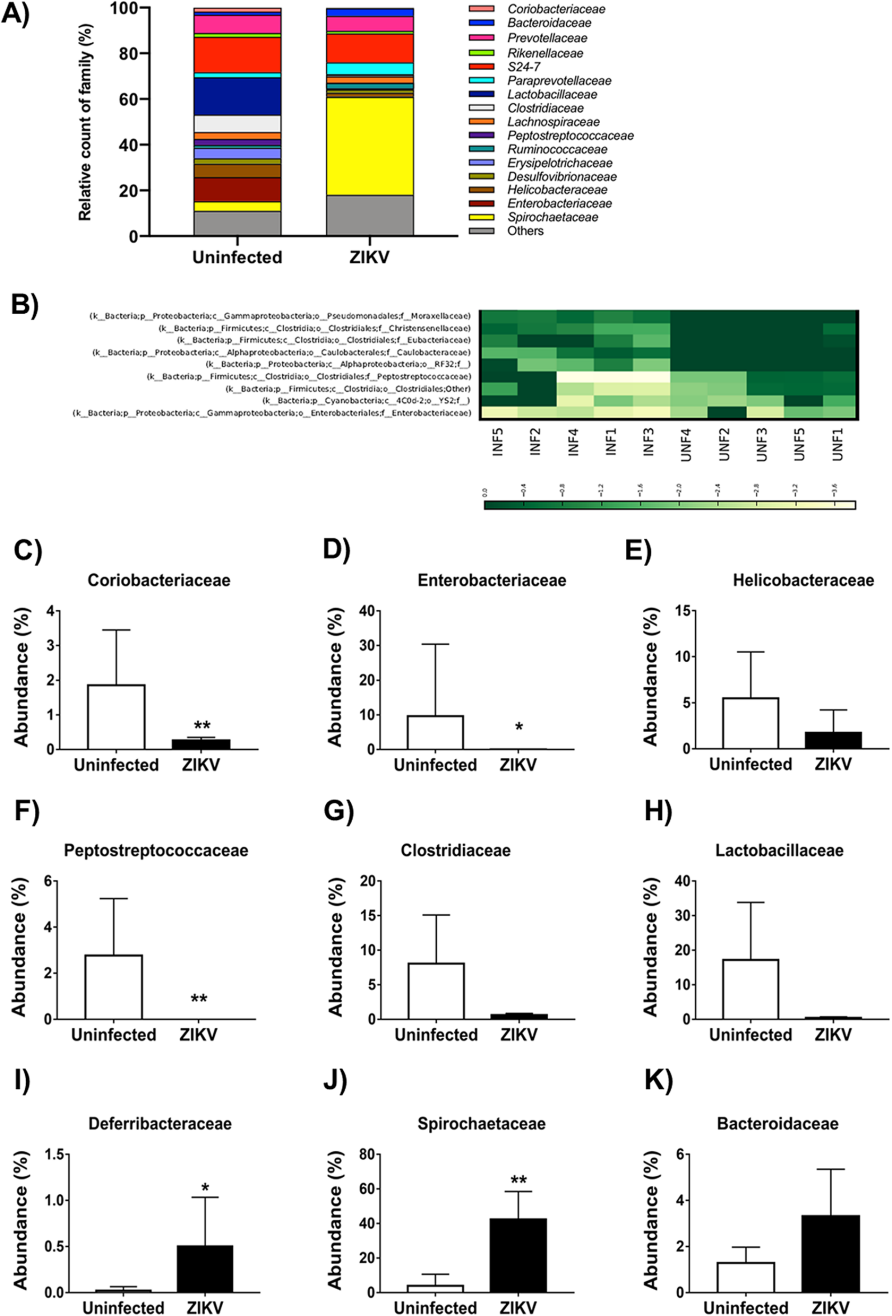
Bacterial families, diversity analysis of the gut microbiota induced by ZIKV infection in the adult immunocompetent mice. C57BL/6J mice (N = 5) were infected with ZIKV and fecal samples were collected after 14 days post-infection. The gut microbiota diversity was assessed by 16S rDNA gene sequencing of the V4 region using the Illumina HiSeq platform. Bar charts represent the relative abundance of Family (**A**), Coriobacteriaceae (**C**), Enterobacteriaceae (**D**), Helicobacteraceae (**E**), Peptostreptococcaceae (**F**), Clostridiaceae (**G**), Lactobacillaceae (**H**), Deferribacteraceae (**I**), Spirochaetaceae (**J**), Bacteroidaceae (**K**). Heat Map (**B**). Statistical analyses were performed using a Student’s two-sample t-test, the non-parametric p-values were calculated with the Mann–Whitney test, the bars represent a confidence interval of 95% (*p < 0.05; **p < 0.01).

At the family level, we also observed an important modulation caused by ZIKV infection in the gut microbiota of the immunocompetent mice (Fig. 3A). The heat map demonstrates the polarization of some family groups (Fig. 3B). The levels of the Coriobacteriaceae, a family within the Actinobacteria phylum, significantly decreased to 0.3% after ZIKV infection in comparison to the uninfected group (1.8%) (Fig. 3C). Similarly, the Enterobacteriaceae family, within the Proteobacteria phylum, significantly decreased in abundance approximately one hundred times in ZIKV-infected mice (0.1%) when compared to uninfected (10.6%) (Fig. 3D). The Helicobacteraceae family, also within the Proteobacteria phylum, seemed to be negatively modulated during ZIKV infection, but not significantly (Fig. 3E). The Peptostreptococcaceae family also decreased its abundance in the ZIKV-infected mice (undetectable, 0%) compared to the uninfected group (2.6%) (Fig. 3F). Also, other families within the Firmicutes phylum, such as the Clostridiaceae (Fig. 3G) and Lactobacillaceae (Fig. 3H), seem to have their abundance negatively impacted on the ZIKV-infected group. However, for these other families, no significant difference relative to the uninfected controls was found.

In contrast, the Deferribacteraceae, a family within the Deferribacteres phylum, increased in the ZIKV-infected group (0.5%) in comparison to the uninfected control group (undetectable) (Fig. 3I). Interestingly, the most drastic impact of ZIKV infection on the gut microbiome of infected immunocompetent mice was on the Spirochaetaceae components, a family within the Spirochaetes phylum, which increased about tenfold (42.9%) relative to the uninfected control group (4.2%; Fig. 3J). Our data did not show any significant modulation of the Bacteroidaceae family in the gut microbiome of the ZIKV-infected mice group (Fig. 3K). Other families were analyzed but were not impacted by ZIKV infection (Table 1).

**Table 1:** Modulation of the bacterial families abundance triggered by ZIKV infection in the adult immunocompetent mice.

Taxonomy	Total	Uninfected	ZIKV
Unassigned;Other;Other;Other;Other	0.2%	0.1%	0.3%
k__Bacteria;p__Actinobacteria;c__Coriobacteriia;o__Coriobacteriales;f__Coriobacteriaceae	1.0%	1.8%	0.3%
k__Bacteria;p__Bacteroidetes;c__Bacteroidia;o__Bacteroidales;f__	0.1%	0.1%	0.1%
k__Bacteria;p__Bacteroidetes;c__Bacteroidia;o__Bacteroidales;f__Bacteroidaceae	2.3%	1.3%	3.3%
k__Bacteria;p__Bacteroidetes;c__Bacteroidia;o__Bacteroidales;f__Porphyromonadaceae	0.3%	0.2%	0.3%
k__Bacteria;p__Bacteroidetes;c__Bacteroidia;o__Bacteroidales;f__Prevotellaceae	7.3%	8.0%	6.6%
k__Bacteria;p__Bacteroidetes;c__Bacteroidia;o__Bacteroidales;f__Rikenellaceae	1.4%	1.7%	1.1%
k__Bacteria;p__Bacteroidetes;c__Bacteroidia;o__Bacteroidales;f__S24-7	14.1%	15.5%	12.7%
k__Bacteria;p__Bacteroidetes;c__Bacteroidia;o__Bacteroidales;f__Odoribacteraceae	0.2%	0.3%	0.1%
k__Bacteria;p__Bacteroidetes;c__Bacteroidia;o__Bacteroidales;f__Paraprevotellaceae	3.7%	2.2%	5.1%
k__Bacteria;p__Cyanobacteria;c__4C0d-2;o__YS2;f__	0.2%	0.0%	0.3%
k__Bacteria;p__Deferribacteres;c__Deferribacteres;o__Deferribacterales;f__Deferribacteraceae	0.3%	0.0%	0.5%
k__Bacteria;p__Firmicutes;c__Bacilli;o__Lactobacillales;f__Lactobacillaceae	8.4%	16.4%	0.4%
k__Bacteria;p__Firmicutes;c__Bacilli;o__Lactobacillales;f__Streptococcaceae	0.1%	0.1%	0.0%
k__Bacteria;p__Firmicutes;c__Clostridia;o__Clostridiales;f__	8.5%	5.7%	11.2%
k__Bacteria;p__Firmicutes;c__Clostridia;o__Clostridiales;f__Clostridiaceae	4.1%	7.6%	0.6%
k__Bacteria;p__Firmicutes;c__Clostridia;o__Clostridiales;f__Dehalobacteriaceae	0.3%	0.3%	0.2%
k__Bacteria;p__Firmicutes;c__Clostridia;o__Clostridiales;f__Lachnospiraceae	2.9%	3.0%	2.9%
k__Bacteria;p__Firmicutes;c__Clostridia;o__Clostridiales;f__Peptostreptococcaceae	1.3%	2.6%	0.0%
k__Bacteria;p__Firmicutes;c__Clostridia;o__Clostridiales;f__Ruminococcaceae	1.9%	1.3%	2.4%
k__Bacteria;p__Firmicutes;c__Erysipelotrichi;o__Erysipelotrichales;f__Erysipelotrichaceae	2.5%	4.6%	0.5%
k__Bacteria;p__Proteobacteria;c__Alphaproteobacteria;o__RF32;f__	0.0%	0.0%	0.1%
k__Bacteria;p__Proteobacteria;c__Alphaproteobacteria;o__Rickettsiales;f__	3.1%	2.6%	3.7%
k__Bacteria;p__Proteobacteria;c__Betaproteobacteria;o__Burkholderiales;Other	0.1%	0.1%	0.1%
k__Bacteria;p__Proteobacteria;c__Betaproteobacteria;o__Burkholderiales;f__Alcaligenaceae	0.9%	0.9%	0.8%
k__Bacteria;p__Proteobacteria;c__Deltaproteobacteria;o__Desulfovibrionales;f__Desulfovibrionaceae	1.9%	2.4%	1.4%
k__Bacteria;p__Proteobacteria;c__Epsilonproteobacteria;o__Campylobacterales;f__Helicobacteraceae	3.7%	5.8%	1.7%
k__Bacteria;p__Proteobacteria;c__Gammaproteobacteria;o__Enterobacteriales;f__Enterobacteriaceae	5.3%	10.6%	0.1%
k__Bacteria;p__Spirochaetes;c__Spirochaetes;o__Spirochaetales;f__Spirochaetaceae	23.5%	4.2%	42.9%
k__Bacteria;p__Tenericutes;c__Mollicutes;o__Mycoplasmatales;f__Mycoplasmataceae	0.1%	0.1%	0.2%
k__Bacteria;p__Tenericutes;c__Mollicutes;o__RF39;f__	0.1%	0.1%	0.1%

### The modulation of gut microbiota induced by ZIKV infection induces leukocyte infiltration in the colon

**Figure 4:**
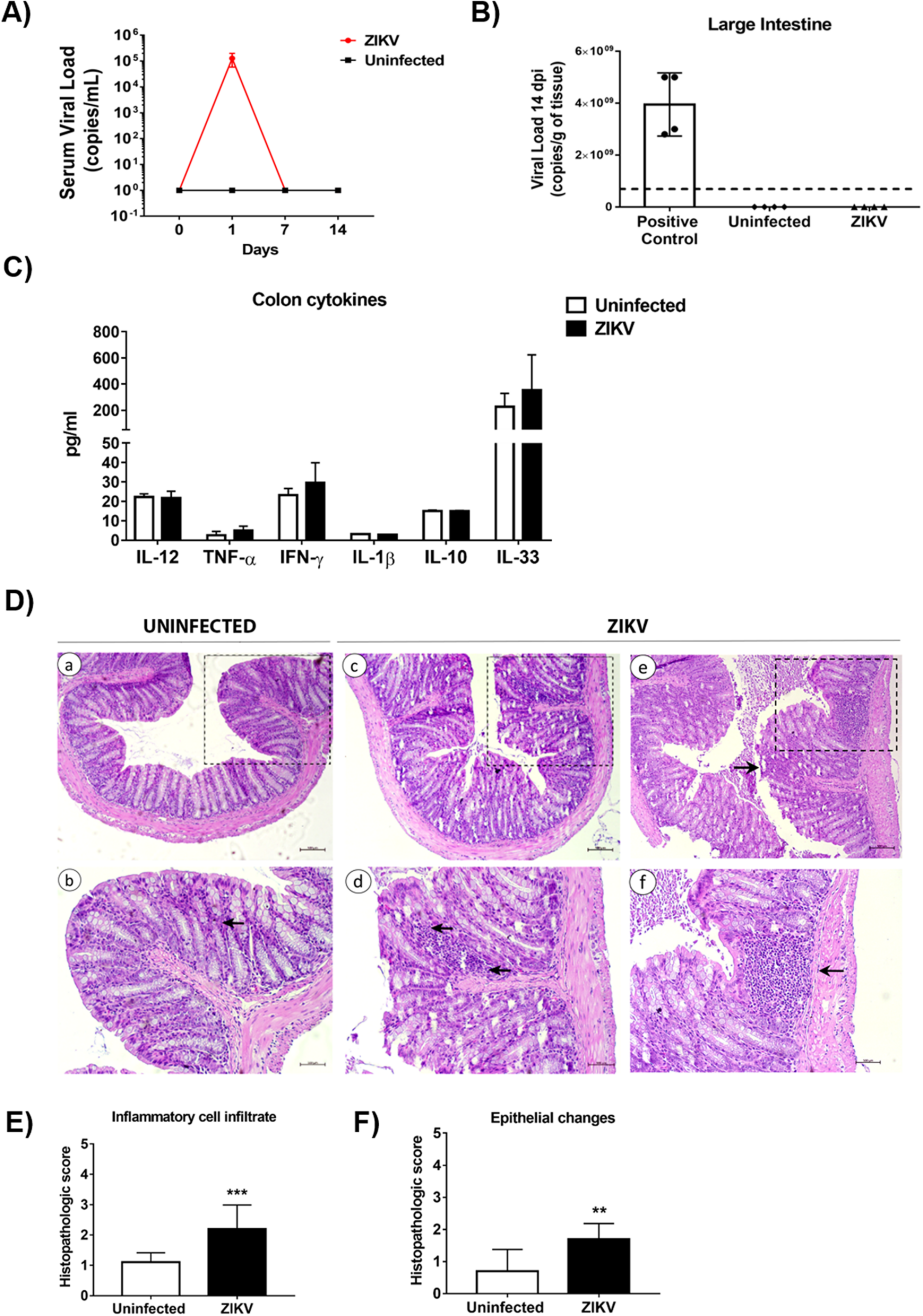
Microbiota changes induced by ZIKV infection cause intestinal epithelium damage and intense leukocyte recruitment to the gut colon in adult immunocompetent mice. C57BL/6J mice (N = 5) were infected with ZIKV and colon tissue was collected after 14 days post-infection. The viral titer was assessed by qPCR in the serum (**A**) and the colon (**B**). The secretion of pro-inflammatory cytokines IL-12, TNF-α, IFN-γ and IL-1β, anti-inflammatory cytokines IL-10, and the cytokine IL-33, was measured by ELISA (**C**). The histological morphology of the colon was analyzed by HE staining and acquired in Zeiss microscope (**D**). Da-b represent section from uninfected mice and Dc-f represent sections from ZIKV infected mice. Db, Dd, and Df present zoomed area from Da, Dc, and De, respectively. Leukocyte infiltration and damaged epithelium are indicated by black arrows. Histological score comparing uninfected and ZIKV mice. The score represents the inflammatory cell infiltration (**E**) and epithelial changes (**F**). Statistical analyses were performed using a Student’s two-sample t-test, the non-parametric p-values were calculated with the Mann–Whitney test. Graphic bars represent a confidence interval of 95% (*p < 0.05; **p < 0.01 ***p < 0.001).

After confirming the changes in the gut microbiota composition induced by ZIKV infection, we asked whether ZIKV could be present in the serum and the gut of mice 14 days post-infection (dpi). To address this question, we performed colon tissue and serum RNA extraction of immunocompetent mice infected or not with ZIKV and performed qPCR. Our results did not demonstrate the presence of ZIKV either in the serum (Fig. 4A) and the colon 14 dpi (Fig. 4B).

Next, we investigated whether ZIKV-induced gut microbiota modulation could induce local inflammation in the intestine. To investigate this, we collected portions of the gut colon of the animals and analyzed both pro-inflammatory and anti-inflammatory cytokine production in this tissue. However, no significant levels of IL-12, TNF-α, IFN-γ, IL-1β, IL-10, and IL-33 were observed between ZIKV-infected and uninfected mice, indicating that ZIKV-induced gut microbiota modulation did not modulate the secretion of these cytokines 14 dpi in immunocompetent mice (Fig. 4C).

Although microbiota modulation induced by ZIKV did not induce any significant alteration in cytokine production in the colon, we detected significant leukocyte infiltration (Fig. 4E) and intestinal epithelium changes (Fig. 4F). HE-stained colon sections displayed moderate inflammatory cell infiltration, which can be seen in mucosa and submucosa of the gut colon, and mild epithelial changes as goblet cell loss, hyperplasia, and erosion on the surface epithelium in the ZIKV-infected mice when compared with uninfected animals (Fig. 4D).

## Discussion

Herein, we showed that wild-type (WT) C57BL/6J male mice presented leukocyte colon infiltration, intestinal epithelial damage, and gut microbiome composition alteration 14 days after ZIKV infection. Although Thackray and colleagues displayed the key roles of intestinal microbiota in host immunity against flaviviruses and suggested intestinal bacterial depletion to be related to exacerbated infection severity^20^, no previous studies have explored the effects of flavivirus infection on the relative abundance of specific bacterial taxa in the mouse gut. Thus, the present work pioneers the investigation of gut microbiome alterations mediated by ZIKV infection of immunocompetent mice.

Human studies informed that ZIKV-infection may affect gastrointestinal (GI) function once some patients reported symptoms such as abdominal pain, nausea, diarrhea, and vomiting^21–23^. Proteomic analyses of ZIKV-infected human colon cells connected this virus with intestinal inflammatory abnormalities and colitis^24^, possibly explaining the reported GI involvement in some ZIKV patients. In contrast, ZIKV is only capable of effectively replicating in mice that show impaired antiviral immune response^25^. This disrupted immunity favors ZIKV survival, which results in a pathology showing intestinal involvements including intestine inflammation^26^ and bowel dilation^27^, alterations that indicate ZIKV as capable of inducing inflammatory processes in the mouse GI tract.

Although immunocompetent animals do not show macroscopic intestinal complications, in this study we detected evidence of inflammatory responses in colon tissue, as we discovered the occurrence of epithelial damage and leukocyte infiltration in the colon of mice 14 dpi. The inflammatory activity has already been linked with gut microbial alterations, as Lupp and colleagues indicated that host-mediated inflammation triggered by infection agents can alter the colonic microbial community^28^. As pro-inflammatory bacteria and pathogens increase their abundance in the gut^29^, increased gut permeability, immune dysfunction, and intestinal epithelial cells damaging are observed^30^. As further detailed here, the communities we discovered to increase in ZIKV-infected mice are related to detrimental health phenotypes. Moreover, bacterial taxa commonly associated with GI tract homeostasis seem to be negatively impacted by the inflammatory responses during the acute phase of infection^29^.

In the present work, we showed that ZIKV infection drastically diminished the abundance of Firmicutes members in the gut microbiome of WT mice. Among the most represented communities in the gut, Firmicutes are the main producers of butyrate^31,32^. This short-chain fatty acid (SCFA) shows anti-inflammatory properties as it impairs leukocyte migration^33^, diapedeses^34^, and enhances intestinal barrier integrity by facilitating tight junction assembly^35^ and influencing mucus production^36^.

Another taxon that dramatically decreased in ZIKV-infected animals is the phylum Actinobacteria. Although less abundant than Firmicutes, Actinobacteria are one of the four major phyla in the intestinal microbiome. They also play key roles in maintaining gut homeostasis through the secretion of SCFAs that protect against enteropathogenic infection^37^, and contribute to intestinal barrier integrity^38^. The fact that members of this phylum are currently used as probiotics highlights their putative beneficial roles for the maintenance of intestinal health^39^.

In contrast, the Deferribacteres and Spirochaetes phyla significantly increased in infected animals, are associated with detrimental pathology in animals^40,41^. Experiments using dextran sodium sulfate (DSS)-treated mice, a colitis animal model, showed that these animals display increased abundance of taxa within the Deferribacteres in the gut compared to untreated animals, strongly associating this phylum with intestinal inflammation^42^. The most severe mouse colitis model, DSS is widely used due to its simplicity and resemblance to human ulcerative colitis (UC)^43^. Although the mechanisms through which DSS affects intestinal homeostasis are not yet fully described, this chemical colitogenic is believed to induce colitis after damaging the epithelial monolayer in the large intestine^44^.

Within the Deferribacteres phylum, *Mucispirillum *sp. (Table 2), enriched in ZIKV-infected mice, is considered an indicator phylotype for DSS treatment^42^. Bacteria from this genus express secretion systems and secrete proteins that modulate intestinal mucosa gene expression, including inflammatory processes^40^. Specifically, the pathobiont *M. schaedleri* is a potent oxygen scavenger, which may enable this species to survive and proliferate in harsh conditions such as inflammatory environments^45^. Also increased in the infected group, the Spirochaetes phylum displayed an increased abundance in the gut when compared with other phyla. As several taxa within the Spirochaetaceae family and *Treponema* genus have been identified as disease-causing^46^ and that an increased presence of *Treponema* in the stool is generally classified as unhealthy^41^, we believe this increase may be relevant for deleterious GI effects during ZIKV infection.

**Table 2:** Modulation of the bacterial genera abundance triggered by ZIKV infection in the adult immunocompetent mice.

Taxonomy	Total	Uninfected	ZIKV
Unassigned;Other;Other;Other;Other;Other	0.2%	0.1%	0.3%
k__Bacteria;p__Actinobacteria;c__Coriobacteriia;o__Coriobacteriales;f__Coriobacteriaceae;g__	0.3%	0.5%	0.1%
k__Bacteria;p__Actinobacteria;c__Coriobacteriia;o__Coriobacteriales;f__Coriobacteriaceae;g__Adlercreutzia	0.7%	1.3%	0.2%
k__Bacteria;p__Bacteroidetes;c__Bacteroidia;o__Bacteroidales;f__;g__	0.1%	0.1%	0.1%
k__Bacteria;p__Bacteroidetes;c__Bacteroidia;o__Bacteroidales;f__Bacteroidaceae;g__Bacteroides	2.3%	1.3%	3.3%
k__Bacteria;p__Bacteroidetes;c__Bacteroidia;o__Bacteroidales;f__Porphyromonadaceae;g__Parabacteroides	0.3%	0.2%	0.3%
k__Bacteria;p__Bacteroidetes;c__Bacteroidia;o__Bacteroidales;f__Prevotellaceae;g__Prevotella	7.3%	8.0%	6.5%
k__Bacteria;p__Bacteroidetes;c__Bacteroidia;o__Bacteroidales;f__Rikenellaceae;g__	0.3%	0.3%	0.3%
k__Bacteria;p__Bacteroidetes;c__Bacteroidia;o__Bacteroidales;f__Rikenellaceae;g__AF12	0.7%	0.7%	0.7%
k__Bacteria;p__Bacteroidetes;c__Bacteroidia;o__Bacteroidales;f__Rikenellaceae;g__Rikenella	0.4%	0.7%	0.2%
k__Bacteria;p__Bacteroidetes;c__Bacteroidia;o__Bacteroidales;f__S24-7;g__	14.1%	15.5%	12.7%
k__Bacteria;p__Bacteroidetes;c__Bacteroidia;o__Bacteroidales;f__[Odoribacteraceae];g__Odoribacter	0.2%	0.3%	0.1%
k__Bacteria;p__Bacteroidetes;c__Bacteroidia;o__Bacteroidales;f__[Paraprevotellaceae];g__Prevotella	3.7%	2.2%	5.1%
k__Bacteria;p__Cyanobacteria;c__4C0d-2;o__YS2;f__;g__	0.2%	0.0%	0.3%
k__Bacteria;p__Deferribacteres;c__Deferribacteres;o__Deferribacterales;f__Deferribacteraceae;g__Mucispirillum	0.3%	0.0%	0.5%
k__Bacteria;p__Firmicutes;c__Bacilli;o__Lactobacillales;f__Lactobacillaceae;g__Lactobacillus	8.4%	16.4%	0.4%
k__Bacteria;p__Firmicutes;c__Bacilli;o__Lactobacillales;f__Streptococcaceae;g__Lactococcus	0.0%	0.1%	0.0%
k__Bacteria;p__Firmicutes;c__Clostridia;o__Clostridiales;f__;g__	8.5%	5.7%	11.2%
k__Bacteria;p__Firmicutes;c__Clostridia;o__Clostridiales;f__Clostridiaceae;Other	0.1%	0.1%	0.0%
k__Bacteria;p__Firmicutes;c__Clostridia;o__Clostridiales;f__Clostridiaceae;g__	3.8%	7.4%	0.2%
k__Bacteria;p__Firmicutes;c__Clostridia;o__Clostridiales;f__Clostridiaceae;g__Clostridium	0.2%	0.1%	0.4%
k__Bacteria;p__Firmicutes;c__Clostridia;o__Clostridiales;f__Dehalobacteriaceae;g__Dehalobacterium	0.3%	0.3%	0.2%
k__Bacteria;p__Firmicutes;c__Clostridia;o__Clostridiales;f__Lachnospiraceae;Other	0.2%	0.2%	0.2%
k__Bacteria;p__Firmicutes;c__Clostridia;o__Clostridiales;f__Lachnospiraceae;g__	2.2%	2.2%	2.2%
k__Bacteria;p__Firmicutes;c__Clostridia;o__Clostridiales;f__Lachnospiraceae;g__Dorea	0.0%	0.0%	0.1%
k__Bacteria;p__Firmicutes;c__Clostridia;o__Clostridiales;f__Lachnospiraceae;g__Ruminococcus	0.5%	0.5%	0.5%
k__Bacteria;p__Firmicutes;c__Clostridia;o__Clostridiales;f__Peptostreptococcaceae;g__	1.3%	2.6%	0.0%
k__Bacteria;p__Firmicutes;c__Clostridia;o__Clostridiales;f__Ruminococcaceae;g__	0.6%	0.5%	0.7%
k__Bacteria;p__Firmicutes;c__Clostridia;o__Clostridiales;f__Ruminococcaceae;g__Oscillospira	1.0%	0.5%	1.4%
k__Bacteria;p__Firmicutes;c__Clostridia;o__Clostridiales;f__Ruminococcaceae;g__Ruminococcus	0.3%	0.3%	0.3%
k__Bacteria;p__Firmicutes;c__Erysipelotrichi;o__Erysipelotrichales;f__Erysipelotrichaceae;g__	0.0%	0.0%	0.1%
k__Bacteria;p__Firmicutes;c__Erysipelotrichi;o__Erysipelotrichales;f__Erysipelotrichaceae;g__Allobaculum	2.4%	4.4%	0.4%
k__Bacteria;p__Firmicutes;c__Erysipelotrichi;o__Erysipelotrichales;f__Erysipelotrichaceae;g__Coprobacillus	0.0%	0.1%	0.0%
k__Bacteria;p__Proteobacteria;c__Alphaproteobacteria;o__RF32;f__;g__	0.0%	0.0%	0.1%
k__Bacteria;p__Proteobacteria;c__Alphaproteobacteria;o__Rickettsiales;f__;g__	3.1%	2.6%	3.7%
k__Bacteria;p__Proteobacteria;c__Betaproteobacteria;o__Burkholderiales;Other;Other	0.1%	0.1%	0.1%
k__Bacteria;p__Proteobacteria;c__Betaproteobacteria;o__Burkholderiales;f__Alcaligenaceae;g__Sutterella	0.9%	0.9%	0.8%
k__Bacteria;p__Proteobacteria;c__Deltaproteobacteria;o__Desulfovibrionales;f__Desulfovibrionaceae;g__	0.4%	0.1%	0.7%
k__Bacteria;p__Proteobacteria;c__Deltaproteobacteria;o__Desulfovibrionales;f__Desulfovibrionaceae;g__Desulfovibrio	1.5%	2.3%	0.6%
k__Bacteria;p__Proteobacteria;c__Epsilonproteobacteria;o__Campylobacterales;f__Helicobacteraceae;g__	0.9%	0.3%	1.5%
k__Bacteria;p__Proteobacteria;c__Epsilonproteobacteria;o__Campylobacterales;f__Helicobacteraceae;g__Flexispira	0.0%	0.0%	0.1%
k__Bacteria;p__Proteobacteria;c__Epsilonproteobacteria;o__Campylobacterales;f__Helicobacteraceae;g__Helicobacter	2.8%	5.5%	0.1%
k__Bacteria;p__Proteobacteria;c__Gammaproteobacteria;o__Enterobacteriales;f__Enterobacteriaceae;g__	5.3%	10.6%	0.1%
k__Bacteria;p__Spirochaetes;c__Spirochaetes;o__Spirochaetales;f__Spirochaetaceae;g__Treponema	23.5%	4.2%	42.9%
k__Bacteria;p__Tenericutes;c__Mollicutes;o__Mycoplasmatales;f__Mycoplasmataceae;g__	0.1%	0.1%	0.2%
k__Bacteria;p__Tenericutes;c__Mollicutes;o__RF39;f__;g__	0.1%	0.1%	0.1%

We hypothesize that ZIKV infection of WT C57BL/6 mice triggers intestinal inflammation during an early phase of the infection that leads to gut dysbiosis, which is characterized by the outgrowth of pathobionts and disruption of beneficial bacterial communities^47^. As we show data that indicate that ZIKV infection favors the outgrowth of bacterial phyla associated with deleterious pathology and drastically reduces bacterial phyla implicated in intestinal barrier integrity and gut homeostasis, we suggest that these alterations may have influenced the occurrence of leukocyte colon infiltration and tissue damage detected by our group.

In this study we did not detect any modulation of colon secreted cytokines; however, microbial communities may influence the secretion of other mediators, such as IL-8 and MCP-1^31,32^. Also, quantification of a broader set of cytokines during the acute phase of ZIKV infection tends to confirm the relevance of the intestinal microbial dysbiosis in this infection. Once immunocompromised mice develop macroscopic intestinal complications, gut microbial analyses of these animals should corroborate the relevance of our gut microbiome data.

Once microbiome composition and function impacts on a plethora of other pathologies^48^, future research should more thoroughly determine the influence of ZIKV infection on this community, as through using female mice or immunodeficient mice, alternative infection routes as subcutaneous or intraperitoneal, other ZIKV strains, and other times of infection. Besides, further experimental efforts should assess GI function as a means of linking microbiome alterations with intestinal involvement, as White and others implicated flavivirus infection with intestinal dysmotility syndromes^27^. Moreover, as oral antibiotic administration was shown to augment mice susceptibility to severe flavivirus infection through interfering with antiviral T cell responses^20^ and possibly exacerbating inflammatory processes^49,50^, employing specific antibiotic treatments must be useful for dissecting the connections between ZIKV infection, dysbiosis, intestinal involvement, and immunity. Uncovering the influence of ZIKV infection on gut microbial communities may prove valuable to determine therapeutic targets for ZIKV infection, a pathology intimately related to the life-threatening conditions of congenital microcephaly^51^ and Guillain–Barré syndrome (GBS)^52^.

**Figure 5:**
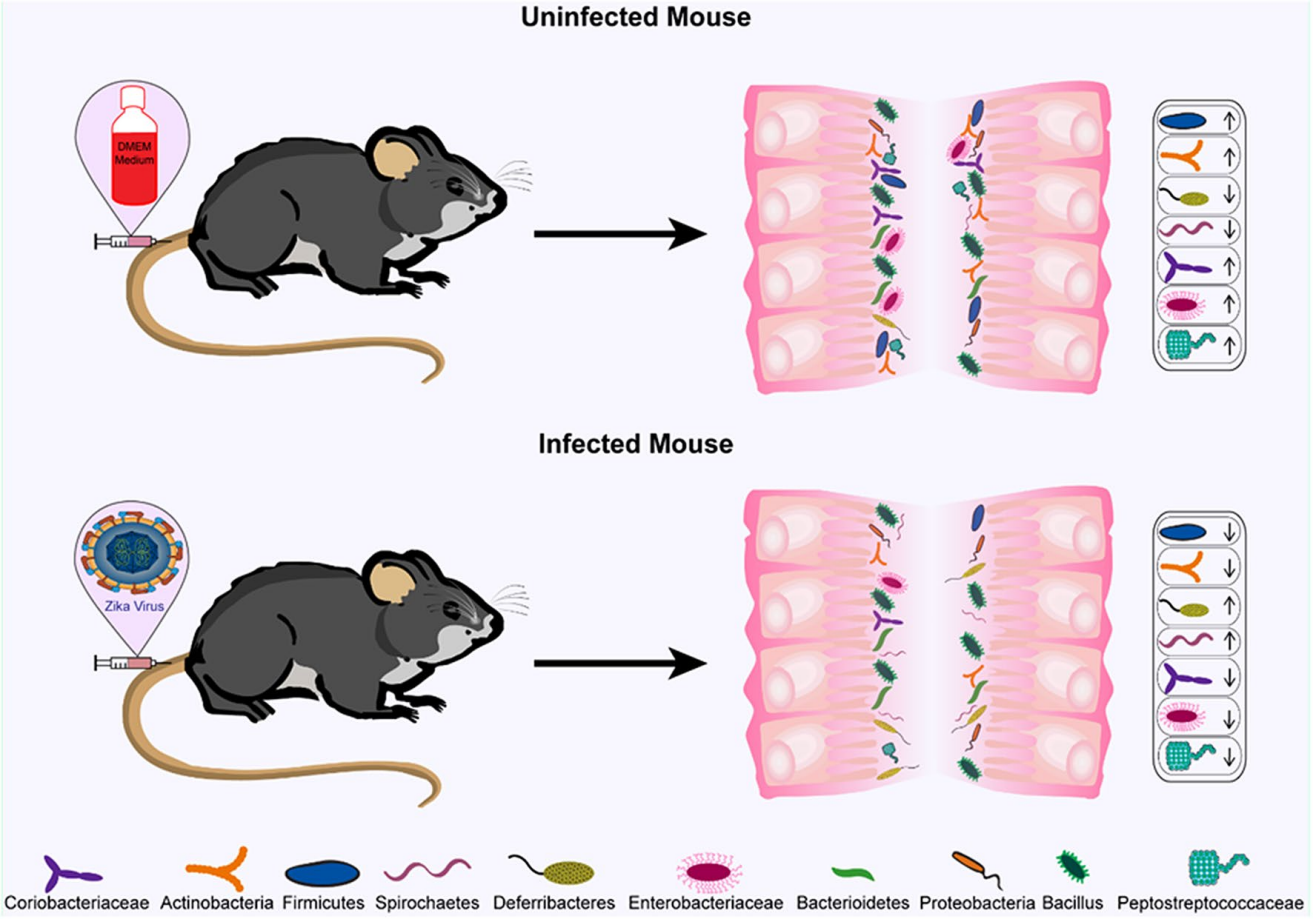
ZIKV infection modulates composition and abundance of specific bacterial taxa from the gut microbiota of the adult immunocompetent mice. ZIKV infected mice (bottom) show significantly altered intestinal microbiota compared to uninfected mice (top). The phyla Proteobacteria, Bacterioidetes, and Bacillus displayed no significant difference between uninfected mice and those infected with ZIKV. ZIKV infection significantly (p < 0.01) decreased the abundance of Firmicutes and Actinobacteria phyla and increased Deferribacteres and Spirochaetes phyla in the gut microbiota of C57BL/6J mice compared to uninfected mice. Moreover, ZIKV infection also triggered a significant decrease of Coreobacteriaceae, Enterobacteriaceae, and Peptostreptococcaceae families (p < 0.05) in gut microbiota.

In conclusion, our study shows for the first time the modulation of the gut microbiota composition by ZIKV in immunocompetent mice (Fig. 5). We show that ZIKV infection decreases the abundance of bacterial families important for the maintenance of gut permeability and gut homeostases, such as Clostridiaceae, Enterobacteriaceae, and Coriobacteriaceae. Besides, ZIKV infection increases the abundance of bacterial families that associate with inflammation, such as Deferribacteraceae, and allows for the emergence of members of pathogenic genera, such as *Treponema* sp*.* Moreover, even though microbiota modulation induced by ZIKV infection did not induce any significant alteration in cytokine production, we observed that microbiota modulation triggered notable leukocyte infiltration and intestinal epithelium damage in the colon of immunocompetent mice infected by ZIKV.

## Methods

### Ethics approval statement

All methods were conducted by relevant guidelines and regulations by CONCEA, Brazil. All experimental protocols in this study were approved by the Committee for Ethics in Animal Use of the Institute of Biological Sciences of the University of Brasilia by protocol number 6117-12. The study was carried out in compliance with the ARRIVE guidelines.

### Animal experiments

Wild-type (WT) C57BL/6J male mice were used at 8–12-week-old. The animals were kept in the Laboratory of Animal Breeding and Experimental Facility of Institute of Biological Sciences of the University of Brasilia, with a 12-h light–dark cycle at controlled temperature (20–25 °C), with food and water ad libitum. The animals were kept in an Alesco (R) filtration system. ZIKV was intravenously injected (i.v) by lateral caudal vein at a viral load of 1 × 10^5^ PFU in a final volume of 100 µL. Dulbecco's Modified Eagle Medium (DMEM) was used as the negative control, with the same final volume injected. A total of 5 animals per group were used in the experiments. After 14 days of infection, feces of both ZIKV-infected and uninfected mice were collected in sterile, RNAse-free centrifuge tubes and frozen at − 80 °C thereafter (the feces were collected directly from the rectum of the animals on the morning of the 14th day after infection). The experiments were repeated at a minimum of 3 times. The animals were euthanized with an overdose of Ketamine Hydrochloride (100 mg/kg) and Xylazine Hydrochloride (100 mg/kg). Subsequently, blood and colon collections were performed.

### Viral stock

For generating the viral stock, the mosquito cell line C6/36 cultured on TC-100 medium supplemented with 2% fetal bovine serum was used (both acquired from Sigma-Aldrich), which was maintained at 37 °C in the absence of CO_2_. Also, we used a Vero cell line (ATCC CCL-81) cultured in Dulbecco’s Modified Essential (DMEM), supplemented with 10% fetal bovine serum, maintained at 37 °C and 5% of CO_2_. ZIKV-PE243 (gene bank reference number KX197192) was kindly provided by Dr. Rafael Freitas de Oliveira França (FIOCRUZ, PE, Brazil) after isolation in 2015 from a human case that occurred in the state of Pernambuco (Brazil). As previously described^53^, the virus was propagated in C6/36 and, after one passage, propagated as well in Vero cells. Stocks were aliquoted and frozen at − 80 °C, where each vial was used a single time. Viral titers were determined by the plaque-forming unit (PFU) and confirmed by RT-qPCR.

### DNA extraction and amplification of V4 region

Mice fecal samples from all groups were collected at the end of the 14 days post-infection and immediately stored at − 80 °C. High-throughput sequencing of the V4 region of the 16S ribosomal DNA (rDNA) gene was performed to characterize the distal gut microbiota composition, according to previously published work^54^. Each sample was subjected to DNA extraction with MoBio PowerSoil DNA isolation kit protocol (MoBio, Carlsbad, CA, USA), before quantification with a Nanodrop. The V4 region of the 16S rRNA gene was PCR-amplified in triplicate with custom barcoded universal bacterial primers using the following protocol: 94 °C for 3 min, 35 cycles of 94 °C for 45 s, 50 °C for 30 s, and 72 °C for 90 s, with a final extension at 72 °C for 10 min^55^, and sequenced on an Illumina HiSeq platform at the Genome Quebec Innovation Center. The quality of amplified material was analyzed on a 1.5% agarose gel, and controls consisted of the PCR reaction without the DNA template. 16S rDNA gene sequences were analyzed using the QIIME software version 1.9.1 package^56^. The sequence data are deposited in the Sequence Read Database (SRA) with accession number SUB7941842.

### Cytokine measurement

The colon from mice was collected, carefully washed with saline, and protein extracted using Lysis Buffer (Tris–HCl 50 mM, NaCl 150 mM, EDTA 5 mM, and Triton-X100 1%) and Cocktail Protease Inhibitor (04,693,159,001, Roche). The expression of IL-12, TNF-α, IFN-γ, IL-1β, IL-10, and IL-33 in the colon were assayed by ELISA according to the manufacturer’s instructions (Mouse TNF-α ELISA Ready-Set-Go, eBioscience, 88–7324–88; Mouse IL-1β ELISA Ready-SET-Go, eBioscience, 88–7013–88; Mouse IL-12/IL-23 total p40 ELISA Ready-SET-Go, eBioscience, 88–7120–88; Mouse IL-10 ELISA Ready-SET-Go 2° Generation, eBioscience, 88–7105–88; Mouse IL-33 DuoSet ELISA kit, R&D Systems, DY3626; Mouse IFN-gamma DuoSet ELISA kit, R&D Systems, DY485).

### Viral load by quantitative RT-PCR

For RNA extraction, 20 mg of colon tissue from infected and uninfected mice was collected and carefully washed with saline. The viral RNA was extracted using the RNeasy Mini Kit (QIAGEN). For better access to the RNA and avoiding inappropriate tissue lysis, TRIzol and chloroform were used before the RNeasy Mini Kit and the top solution phase was driven to the next viral RNA isolation step, with the above-mentioned kit. After RNA isolation and purification following the manufacture’s instruction, viral load was accessed by one-step quantitative reverse transcriptase PCR (RT-q PCR) as previously described^53^. A published primer set was used to detect ZIKV RNA^57^: Fwd, 5′-CCGCTGCCCAACACAAG-3′; Rev, 5′-CCACTAACGTTCTTTTGCAGACAT-3′; Probe, 5′-FAM/AGCCTACCTTGACAAGCAGTCAGACACTCAA/3IABkFQ/-3′ (Integrated DNA Technologies). ZIKV antigens produced in immunocompromised mice brain was provided by the Central Laboratory of Federal District and was used as a positive control.

### Histology analysis

The colon from mice was collected, carefully washed with saline buffer, and fixed in 10% Formalin. Thereafter, the tissue was cut into slices, dehydrated, and embedded with paraffin. The slides were stained with hematoxylin and eosin (HE) (Sigma) following standard procedures^58^. Sections were examined by light microscope Zeiss Lab. A1 Axiocam 105 color and photomicrographs were scanned using the ZEN program from Zeiss. Tissue samples were well-oriented with longitudinally cut crypts to precisely assess alterations in the overall intestinal tissue architecture. The slides were blindly scored based on a semiquantitative scoring system that includes the main alterations observed: (I) inflammatory cell infiltrate: severity and extent, (II) Epithelial changes: hyperplasia, goblet cell depletion, and erosion. Each parameter could receive 0–5 in the score index (0: normal; 1: minimal; 2: mild; 3: moderate; and 4 or 5 extensive)^59^.

### Statistical analysis

Results contained in the present work were reported by mean ± standard deviation (SD). Statistical differences among the two compared groups were made using Student’s t-test with Bonferroni corrections provided by GraphPad PRISM Software version 6.00 or QIIME software version 1.9.1. p values are represented by asterisks: p ≤ 0.05 (*), p ≤ 0.01 (**), p ≤ 0.001 (***) and p ≤ 0.0001 (****).

## Supplementary Information

Supplementary Information.

## Data Availability

Sequence data are deposited in the Sequence Read Database (SRA) with the accession number SUB7941842.
