# Supplementary material for: Gut microbiota modulation induced by Zika virus infection in immunocompetent mice
Source: Sci Rep. 2021 Jan 14;11:1421. doi: 10.1038/s41598-020-80893-y (PMC7809017; doi:10.1038/s41598-020-80893-y)
Supplement: Supplementary file 1 — Supplementary Information. [file 41598_2020_80893_MOESM1_ESM.docx]

**Gut microbiota modulation induced by Zika virus infection in immunocompetent mice**

Rafael Corrêa^1^, Igor de Oliveira Santos^1^, Heloísa Antoniella Braz-de-Melo^1^, Lívia Pimentel de Sant’Ana^1^, Raquel das Neves Almeida^1^, Gabriel Pasquarelli-do-Nascimento^1^, Paulo Sousa Prado^2^, Gary P. Kobinger^3,4^, Corinne F. Maurice^5^ and Kelly Grace Magalhães^1*^


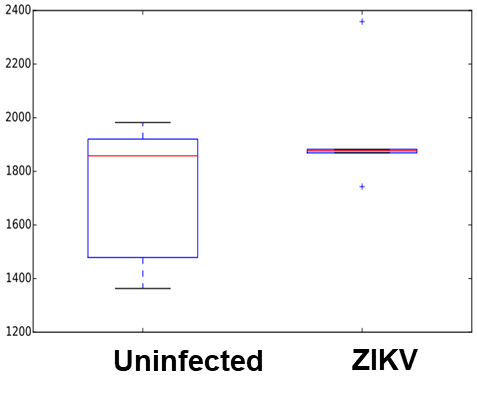


**Supplementary Figure 1:** α-Diversity of Gut Microbiota. Analysis of alpha diversity predicted diversity by Chao1estimator in Uninfected vs. ZIKV, p = 0,222.
